# Supplementary material for: The SHED Index: A Validation Study to Assess Sustainable HEalthy Diets in Portugal
Source: Nutrients. 2023 Dec 12;15(24):5071. doi: 10.3390/nu15245071 (PMC10745297; doi:10.3390/nu15245071)
Supplement: Supplementary file 1 [file nutrients-15-05071-s001.zip › nutrients-2721338-supplementary.pdf]

**Supplemental Materials**

**Table S1.** Cronbach's alpha and item-total correlation.

| | Corrected Item-Total Correlation | Cronbach's Alpha if Item Deleted | Global Cronbach's Alpha |
|---|---|---|---|
| **Healthy eating score** | | | 0.820 |
| healthy.eat.1 | 0.381 | 0.818 | |
| healthy.eat.2 | 0.477 | 0.807 | |
| healthy.eat.3 | 0.495 | 0.805 | |
| healthy.eat.4 | 0.533 | 0.801 | |
| healthy.eat.5 | 0.535 | 0.800 | |
| healthy.eat.6 | 0.530 | 0.801 | |
| healthy.eat.7 | 0.373 | 0.816 | |
| healthy.eat.8 | 0.635 | 0.790 | |
| healthy.eat.9 | 0.496 | 0.805 | |
| healthy.eat.10 | 0.591 | 0.795 | |
| **Sustainable eating score** | | | 0.704 |
| sustainble.eating.1 | 0.334 | 0.699 | |
| sustainble.eating.2 | 0.460 | 0.659 | |
| sustainble.eating.3 | 0.419 | 0.669 | |
| sustainble.eating.4 | 0.525 | 0.641 | |
| sustainble.eating.5 | 0.529 | 0.640 | |
| sustainble.eating.6 | 0.364 | 0.684 | |
| sustainble.eating.7 | 0.302 | 0.698 | |
| **BFV location score** | | | 0.477 |
| Fruits.vegetables.1 | 0.186 | 0.458 | |
| Fruits.vegetables.2 | 0.203 | 0.447 | |
| Fruits.vegetables.3 | 0.166 | 0.461 | |
| Fruits.vegetables.4 | 0.196 | 0.452 | |
| Fruits.vegetables.5 | 0.361 | 0.372 | |
| Fruits.vegetables.6 | 0.380 | 0.374 | |
| Fruits.vegetables.7 | 0.250 | 0.441 | |
| Fruits.vegetables.8 | 0.015 | 0.520 | |
| **Ready-meals score** | | | 0.432 |
| ready.meals.1 | 0.298 | 0.340 | |
| ready.meals.2 | 0.230 | 0.378 | |
| ready.meals.3 | 0.215 | 0.386 | |
| ready.meals.4 | 0.203 | 0.395 | |
| ready.meals.5 | 0.182 | 0.404 | |
| ready.meals.6 | 0.154 | 0.427 | |
| **Water score** | | | 0.409 |
| Water.1 | 0.279 | 0.292 | |
| Water.2 | 0.048 | 0.498 | |
| Water.3 | 0.140 | 0.405 | |
| Water.4 | 0.502 | 0.080 | |
| Water.5 | 0.144 | 0.399 | |
| **Sodas score** | | | 0.471 |
| Soft.drinks.1 | 0.298 | 0.369 | |
| Soft.drinks.2 | 0.266 | 0.435 | |

| | | |
|---|---|---|
| Soft.drinks.3 | 0.358 | 0.329 |

BFV—fruits and vegetable purchasing location.
